# Enhancing an online cognitive behavioural therapy intervention for depression: Harnessing the feedback of sexual and gender minority youth to help improve SPARX

**DOI:** 10.1177/10398562231153061

**Published:** 2023-01-23

**Authors:** Mathijs FG Lucassen, Karolina Stasiak, Theresa Fleming, Matthew Shepherd, Sally N Merry

**Affiliations:** Department of Health and Social Care, 5488 The Open University, Milton Keynes, UK; Department of Psychological Medicine, 1415 The University of Auckland, Auckland, New Zealand; Department of Psychological Medicine, 1415 The University of Auckland, Auckland, New Zealand; School of Health, 8491 Victoria University of Wellington, Wellington, New Zealand; Department of Psychological Medicine, 1415 The University of Auckland, Auckland, New Zealand; School of Psychology, 168219 Massey University – Albany Campus, Auckland, New Zealand; Department of Psychological Medicine, 1415 The University of Auckland, Auckland, New Zealand

**Keywords:** bisexual, depression, gay, lesbian, transgender

## Abstract

**Objective:**

SPARX is an online cognitive behavioural therapy self-help intervention for adolescent depression provided in serious game format. Since 2014, it has been freely available in Aotearoa New Zealand (NZ) due to funding from the NZ government. In 2020/21, feedback from sexual and gender minority youth (SGMY) was used to refine and update SPARX.

**Method:**

Three online focus groups and follow-up email consultations involved 12 SGMY (16 to 25 years old) in NZ. A general inductive approach was used to analyse data.

**Results:**

SGMY had specific needs as well as preferences and four themes were identified: attend to our contextual realities; portrayals of sexual and gender minority people in games; envisaged ideals for serious gaming and appraisals of SPARX. SGMY feedback was used to improve SPARX for this unique population, with the updates launched in October 2021.

**Conclusions:**

SGMY are underserved in terms of their mental health needs. Refining or tailoring existing interventions proffers a potential way forward in terms of addressing these needs.

Sexual minority (e.g. lesbian, gay and bisexual) youth are at an increased risk of depression,^1^ with gender minority (e.g. transgender/trans) youth having particularly high mental health needs.^2^ Unfortunately, sexual and gender minority youth (SGMY) are frequently stigmatised and mistreated and, as a result, they are more likely to experience compromised mental health.^3^ Limited intervention options specific to SGMY are available;^4^ however, prior research indicates that these young people value the online delivery of psychological supports for their mental health.^4^ One such online intervention, SPARX (Smart, Positive, Active, Realistic, X-Factor thoughts) is provided in serious game format; it has been used by over 9000 adolescents and evaluated amongst SMGY for almost a decade.^2,5^ Serious games (in brief, digital games for health, education or another ‘serious’ purpose) may be useful for increasing the reach and impact of online interventions given the popularity and non-threatening nature of games.^6^ However, results to date indicate that this seven-module online cognitive behavioural therapy intervention should be refined with SGMY in mind.^4,5^ SPARX has been made freely available in Aotearoa New Zealand (NZ) since 2014 with 2.3% of its users (*n* = 207) identifying as transgender.^2^ In 2020, funding from the NZ Ministry of Health was obtained to update the intervention. As part of the co-design work associated with this upgrade, we obtained additional funding and sought the input of SGMY. In this small qualitative study, we asked SGMY to draw upon their experiences of SPARX, and computer games more generally, to consider:

• In which ways should a serious game for mental health, like SPARX, be refined so that it better meets the needs of SGMY?

## Methods

The consolidated criteria for reporting qualitative research (COREQ)^7^ has informed this study’s write-up.

### Sample and data collection

Participants were recruited via a staff member that was known to them from a SGMY organisation in NZ, whereby staff posted online messages about the study and those young people that expressed an interest in it were sent additional information. This information included online promotional material about the study, and this was sent to those aged 16–25 years old that were keen to take part. The participants were from one of two SGMY organisations based in major cities. However, they came from a range of locations in the North Island of New Zealand.

### Ethics and consent

Ethics approval was granted by the Health and Disability Ethics Committee in NZ. All participants provided written informed consent prior to taking part in the study.

### Focus groups

Three online focus groups (*N* = 12) consisted of ten participants aged 16–19 years and two participants aged 20–25 years; all participants were SGMY (see Table 1). Focus groups comprised of the participants (in private spaces in various locations) and ML, who facilitated all the sessions. These groups lasted between 59 and 65 minutes. A semi-structured format (questions available from the corresponding author) was used. Initial questions (and prompts) focused on participants’ experiences of games generally and then (after a short demonstration of SPARX) questions about this serious game, for example:

• Do you play computer games, if so, which ones [prompts – examples of games on smartphones, games played alone versus with others and well-known games versus those not so many people know about]?
• Have you used the program called SPARX? If yes, what did you think of it? If no, would you consider using a computer/digital program to help you if you were feeling stressed or low?

**Table 1.** Demographics and details about the participants

	Pseudonym	Age range	Ethnicity	Gender (pronouns) and sexuality^a^	Plays computer games	Have used SPARX
Focus Group 1	Dahlia	20–25 years old	NZ European	Female^b^ (she/her) and pansexual	Yes	Yes
	Bella	16–19 years old	NZ European	Nil response (she/her) and nil response	No	Yes
	Cobi	16–19 years old	NZ European	Nil response (they/them) and stated ‘other’^c^	Yes	Yes
	Drew	20–25 years old	European heritage^c^	Non-binary (they/he) and stated ‘other’^c^	Yes	Yes
Focus Group 2	Carter	16–19 years old	European heritage^c^	Trans (they/them) and queer	Yes	Yes
	Rylee	16–19 years old	Asian heritage^c^	Male^b^ (he/him) and mostly heterosexual	Yes	Yes
	Nat	16–19 years old	Māori and NZ European	Non-binary (they/them) and gay/lesbian	Yes	Yes
	Keegan	16–19 years old	NZ European	Male^b^ (he/him) and bisexual	Yes	Yes
Focus Group 3	Kylie	16–19 years old	NZ European	Female^b^ (she/her) and bisexual	Yes	No^d,e^
	Evelyn	16–19 years old	NZ European	Female (she/her) and ‘other’^c^	Yes	No^e^
	Kim	16–19 years old	NZ European	Agender (they/them) and ‘other’^c^	No	No^e^
	Blake	16–19 years old	Māori and NZ European	Male (he/him) and gay	Yes	No^e^

^a^Some participants did not answer certain questions in the demographic questionnaire.

^b^Participants’ gender was different from their sex assigned at birth.

^c^To protect the identity of the participant, we have deliberately avoided providing further elaboration.

^d^Had not used SPARX but in the past had been recommended it.

^e^Had not used SPARX but had heard of it and engaged with demonstration.

Subsequent questions focused on more specific details, for instance, if participants could change only one thing in relation to SPARX for SGMY, what it would be. After the focus groups, participants provided feedback via email regarding the game development company’s prototypes or planned refinements for SPARX.

The facilitator (ML), who is based in the United Kingdom, is an academic experienced in clinical and group-based youth mental health work. The participants had not previously met ML, but they were aware that he was a co-developer of SPARX. They also knew ML is a gay male and gender role non-conformer, and participants seemed at ease knowing this about him. Focus groups were audio-recorded and professionally transcribed. Basic field notes were taken.

### Data analysis

We used a general inductive approach (GIA) for data analysis.^8^ GIA is a method of content analysis which seeks to build understandings from participants’ comments on a specific research question (or questions) as opposed to testing pre-existing hypotheses. The transcripts were read with the research question in mind. ML read and re-read the transcripts and identified lower order units of meaning which were then clustered with similar units. Units were reduced to address overlap and redundancy among the categories before the final themes and sub-themes were agreed. A preliminary summary of results was sent to participants.

## Results

Twelve SGMY participated, and a further potential participant wanted to take part but could not do so given the timings of the focus groups. Almost all (10/12, 83%) identified as gamers and two-thirds (8/12, 67%) had used SPARX. The remaining participants (4/12 – all in Focus Group 3) who had not used SPARX engaged with the short demonstration of the program during their focus group. Table 2 summarises the results of the focus groups.

**Table 2.** Summary of focus group results

Major theme	Associated sub-themes	Descriptions and examples from the data
Attend to our contextual realities	Sexual and gender minority people are hidden away or stigmatised	Participants in their day-to-day lives, and whilst gaming, recognised issues to do with SGMY being hidden away or stigmatised. For example, Cobi noted attitudes in a gaming context where ‘…we can’t show that, that’s gross, ew, men being gay…’ Whilst Rylee stated ‘…because rainbow [i.e., sexual and gender minority] erasure is very real, it is a thing that we struggle with a lot…’ and Kylie said: ‘…it’s relatively uncommon to find same sex couples in games and I don’t think I’ve ever played a game where being trans is an option’.
	Negative reactions to a sexual and gender minority presence	Negativity occurred in real life and whilst gaming. For instance, Evelyn mentioned ‘Like it is very much still normalised, homophobia, and we live in [small city in the North Island] and I’ve still experienced people on the street being like, you’re wearing something rainbow, oh my God, I’m going to call you a slur…’ Drew noted in gaming: ‘…which is very much dominated by cishet [cisgender/not transgender heterosexual] white guys, who at even the slightest sniff of change in representation in games [i.e., sexual and gender minority inclusion]…they just lose it’.
	Internalisation of negative messages	‘Tragic’ ways in which sexual and gender minority people are presented in games (and elsewhere) can result in a young person believing ‘…oh, I’m different, oh, I have so much hardship…it just makes people feel bad that being any different is bad, like just gender, bigender or whatever, like just being queer in general…’ (Cobi). Or Kim noted: ‘…like I know a lot of gay people and stuff are like, some of their immediate thoughts when they’re just realising it, it’s like, oh gosh, I’m not normal, or, it’s not okay, I’m not going to be accepted…’
	Games and gaming are valuable	Amongst participants (10/12 described themselves as gamers) gaming was important for a range of reasons. For Bella, it was a valuable distraction ‘…I use gaming as a, here’s a fun thing to make you stop thinking about all the bad things…’ Or Carter found a particular game that is ‘really important’ as it ‘…is a really happy and relaxing thing’. Whereas Evelyn noted ‘I find playing [games] with a group of friends helps [to create safer environments]’.
Portrayals of sexual and gender minority people in games	Minorities are under-represented	Participants noted that games in general do poorly in terms of representation (e.g. exclude people with disabilities), as Drew mentioned ‘…minorities are still very under-represented [in games]…’ When sexual and gender minority people are included, this is often tokenistic, such as ‘…just having a footnote on a character’s bio that they’re trans [transgender]’ (Dom).
	Depicted badly or negatively	When depicted, portrayals are frequently done ‘really badly’, as Carter noted as an example from a game: ‘…but it’s quite sad, especially like there’s a trans [transgender] boy in it and his entire pain comes from being trans…so it’s representation but it’s sad representation’. When included, it’s often stereotypically so, as Bella noticed in ‘…we don’t have representation, or you get games that are entirely focused on that and then…’ - Cobi interjects: ‘They have the openly effeminate guy’.
	Gaming done well	Participants also cited examples of gaming done well, such as The Sims, Journey and Dream Daddy. Dahlia explained: ‘Call of Duty: Cold War…has an option to have your character use they/them pronouns…it’s very open and inclusive’. Drew highlighted: ‘…look at indie [independently developed] games made by queer developers you can find some absolutely wonderful games that are very much orientated around being a queer person…’
Envisaged ideals for serious gaming	Move beyond the binary	Options beyond a male/female binary are recommended in games: ‘I don’t like them [the avatars in SPARX]! One of them is very like, I’m a man, and one of them is like, I have boobs, I’m a woman. I wish things were a bit more androgynous I guess’ (Cass) and ‘…get rid of having two avatars [i.e., in SPARX] and just give options to change certain parts of the avatar…’ (Carter). Or as Kylie recommended: ‘Just make it a bit more accessible to give more masculine body shapes a dress or more feminine body shapes a suit just so you can create characters that fall in between a bit more’.
	Abilities to explore and make sense of gender (or sexuality)	Opportunities for exploration were recognised as important, as Rylee noted: ‘…a big part of being…this minority [i.e., SGMY] is that you constantly change, you learn new information and you constantly change’. For instance, being able to pick and change gender pronouns allowed for exploration: ‘…if the pronouns were used and they were able to be changed, then it’s a cool way for trans people or questioning people or anybody like that to try out pronouns without having to endanger themselves or do anything else…’ (Keegan).
	Contemporary gameplay and experiences expected	Serious games like SPARX should be ‘updated’, technical issues need to be addressed (e.g. ‘I could barely play it on my phone’ Cobi) and serious games should compare well to commercial products. As Dahlia noted: ‘…I felt that [SPARX] was a bit too short for what I feel like people who engage in games would be expecting to play’ And Rylee explained: ‘…games need to be more interactive with the person, because for games like first-person shooter games like Call of Duty, it’s constantly you talking with your other mates…’
Appraisals of SPARX	Format and approach of SPARX problematic	Participants noted issues to do with the format and approach adopted in SPARX, such that there was too much text/reading in SPARX: ‘…I just came across the breathing technique [in SPARX] and I think having it as a text just is a very ineffective way to show it off…’ (Nat). Cobi stated: ‘…it’s just the mechanics are confusing, it’s a bit cliché as well, because the first level is like a chest which says, hope lies within, and then a bird called Hope comes out and then someone’s like, oh, is that the Hope bird, I thought we had lost it!’
	SPARX should be more inclusive	Inclusion issues were identified regarding SPARX, with Cobi saying: ‘…the game doesn’t feel really inclusive because most of the people are just skinny and light skinned’. Body-size issues were also reinforced by Kim: ‘Yeah, like body types, like from what I’ve seen they’re all really skinny and not everyone’s skinny!’ However, a Māori participant (Blake) noticed: ‘…it’s quite cool because it’s like Māori and they have on some of the avatars…they have like designs and the pounamu [a carved greenstone] one of them is wearing, so it’s quite cool’.
	SPARX can be appealing	There was no clear consensus in terms of SPARX’s appeal, as some participants did not find it engaging or anticipated that it would only be useful after it was sufficiently updated and modified. However, several participants did think SPARX was appealing. As Carter said: ‘I think overall it’s a really good way of giving people the techniques to listen with stuff that they’ve got going on and I acknowledge that it’s not meant to be like a therapist’.

## Discussion

**Figure 1.**
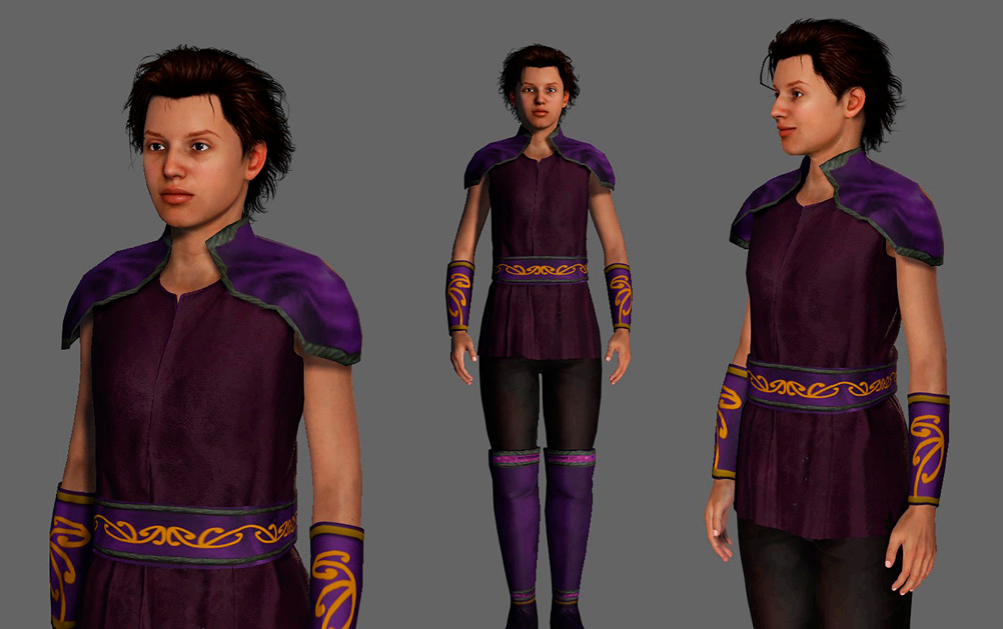
New avatar (in addition to the ‘male’ and ‘female’ options previously available in SPARX). Image used with permission of the copyright owner © Auckland UniServices Limited.

Building on earlier work, we had the opportunity to enhance SPARX, in consultation with SGMY. Prior research has already focused on improving the intervention for Māori users [e.g.^9^], and exploratory research has begun amongst young people that have a long-term physical health condition.^10^ However, feedback from SGMY suggested that the intervention be updated as it was ‘*old fashioned*’. Participants also provided specific ideas about how SPARX could be made more acceptable to SGMY. Focus group findings and follow-up email consultations after the focus groups led to several changes being made to create the updated version of SPARX (see Table 3 and Figure 1 for details).

**Table 3.** Summary of recommendations from SGMY and the corresponding results

Recommendations from SGMY	An existing feature of SPARX	Changes funded by NZ Ministry of Health	Changes funded by current study	Changes not possible for the updated version
**Specific suggestions about SPARX’s avatar**				
Move beyond the binary (solution – an androgynous option)			✓	
Customisation options the same regardless of avatar selected	✓			
Ability to change the gender of the avatar once selected				✓^b^
‘Gender slider’ (from a very feminine to very masculine avatar)				✓^b^
Enhance diversity so the avatar can be made ‘plus-sized’				✓^b^
Select avatar’s pronouns (e.g., she/her or they/them)			✓	
**Specific suggestions about SPARX’s gameplay**				
Ability to reload (or replay) levels/modules in SPARX		✓		
SPARX to be ‘polished’ (e.g. update the graphics)		✓		
Create SPARX minigames that aren’t just fighting ‘Gnats’^a^				✓
Make SPARX a multiplayer game				✓
Make SPARX work better on phones (and other devices)		✓		
Make SPARX easier to download (and access)		✓		
Reduce dialogue and make it ‘less like reading from a book’		✓		
Enhance the game controls (e.g. ability to use a mouse)				✓

^a^Glooming Negative Automatic Thoughts (Gnats).

^b^Changes we would prioritise for SGMY if we had been given additional funding.

Earlier work has already investigated the importance of identity-affirming avatars in gaming for gender minority youth.^11^ Similarly, we also found that games can provide valuable opportunities for young person to explore their gender identity, and yet gaming often constrains diverse gender expression, an issue that can be overcome with enhanced customisation options.^11^ We have attempted to address some of the issues raised by SGMY but did not have sufficient funds to address these issues as fully as we would have liked.

Strengths of this study include using an established qualitative method to help improve a freely available evidence-based psychotherapy together with SGMY – an underserved population. Notable limitations include this being a small-scale exploratory study which only recruited from SGMY-specific organisations. ML, who facilitated the focus groups, is part of the development team that created SPARX and participants knew this, and as a result, some participants may have felt inhibited to express criticisms of the program. Recruitment was challenging, as is often the case for studies focused on this unique population^12^ and even more so during a global pandemic. We attempted to include intersex young people, given earlier SPARX-related work identified their elevated mental health needs,^13^ but unfortunately, we were unsuccessful in this endeavour. Ideally, more online focus groups would have taken place; however, given the potential issues related to research fatigue associated with marginalised populations^14^ and the time pressures to launch the updated version of SPARX, this was not possible. Despite these factors, this study is worthwhile because it has sought to listen to the voices of a marginalised group so that SPARX better meets the needs of SGMY; this listening to underserved populations is an important initial step towards a necessary process of change.^15^

## Conclusion

SGMY are more likely to experience mental ill-health and interventions such as SPARX can make a difference to their wellbeing. We have demonstrated that SGMY have valuable insights and perspectives which can help enhance the acceptability of tools such as SPARX for SGMY. It is important to respond to the needs of SGMY, and this can be done by better tailoring ‘mainstream’ interventions. Ongoing monitoring of the uptake of SPARX will be useful to establish if we have increased engagement in terms of this group and other important underserved populations (e.g. Māori rangatahi/adolescents and young people with long-term physical health conditions).

## References

[1] Lucassen MFG Stasiak K Samra R , et al. Sexual minority youth and depressive symptoms or depressive disorder: A systematic review and meta-analysis of population-based studies. Australian & New Zealand Journal of Psychiatry 2017; 51: 774–787. 28565925 10.1177/0004867417713664

[2] Lucassen MFG Stasiak K Fleming T , et al. Computerized cognitive behavioural therapy for gender minority adolescents: Analysis of the real-world implementation of SPARX in New Zealand. Australian and New Zealand Journal of Psychiatry 2021; 55: 874–882. 33287554 10.1177/0004867420976846 PMC8404718

[3] Meyer IH . Prejudice, social stress, and mental health in lesbian, gay, and bisexual populations: Conceptual issues and research evidence. Psyc Bulle 2003; 129: 674–697. 10.1037/0033-2909.129.5.674 PMC2072932 12956539

[4] Lucassen MFG Samra R Iacovides I , et al. How LGBT+ young people use the internet in relation to their mental health and envisage the use of e-therapy: Exploratory study. JMIR Serious Games 2018; 6: e11249. 30578194 10.2196/11249 PMC6320432

[5] Lucassen MFG Hatcher S Fleming TM , et al. A qualitative study of sexual minority young people’s experiences of computerised therapy for depression. Australasian Psychiatry 2015; 23: 268–273. 25881962 10.1177/1039856215579542

[6] Fleming TM Bavin L Stasiak K , et al. Serious games and gamification for mental health: Current status and promising directions. Frontiers in Psychiatry 2017; 7: e215. 10.3389/fpsyt.2016.00215 PMC5222787 28119636

[7] Tong A Sainsbury P Craig J . Consolidated criteria for reporting qualitative research (COREQ): a 32-item checklist for interviews and focus groups. Int J Quali Health Care 2007; 19: 349–357. 10.1093/intqhc/mzm042 17872937

[8] Thomas DR . A general inductive approach for analyzing qualitative evaluation data. Am J Evalu 2006; 27: 237–246.

[9] Shepherd M Fleming T Lucassen M , et al. The design and relevance of a computerized gamified depression therapy program for indigenous Māori adolescents. JMIR Serious Games 2015; 3: e1. 25736225 10.2196/games.3804 PMC4392467

[10] Thabrew H Stasiak K Garcia‐Hoyos V , et al. Game for health: How eHealth approaches might address the psychological needs of children and young people with long‐term physical conditions. Journal of Paediatrics and Child Health 2016; 52: 1012–1018. 27529150 10.1111/jpc.13271

[11] Morgan H O’Donovan A Almeida R , et al. The role of the avatar in gaming for trans and gender diverse young people. Int J Environ Res Public Health 2020; 17: e8617. 10.3390/ijerph17228617 PMC7699515 33233536

[12] Lucassen MFG Fleming TM Merry SN . Tips for research recruitment: The views of sexual minority youth. Journal of LGBT Youth 2017; 14: 16–30.

[13] Lucassen MFG Perry Y Frampton C , et al. Intersex adolescents seeking help for their depression: the case study of SPARX in New Zealand. Australasian Psychiatry 2021; 29: 450–453. 33626301 10.1177/1039856221992642 PMC8361470

[14] Ashley F . Accounting for research fatigue in research ethics. Bioethi 2021; 35: 270–276. 10.1111/bioe.12829 33205395

[15] Shevlin M Rose R . Respecting the voices of individuals from marginalised communities in research—“Who is listening and who isn’t?”. Edu Science 2022; 12: 304.

